# Reliability and validity testing of the medicines related - consultation assessment tool for assessing pharmacists’ consultations

**DOI:** 10.1007/s11096-022-01489-2

**Published:** 2022-11-17

**Authors:** Helen Middleton, Lesley Grimes, Sarah C. Willis, Douglas Steinke, Matthew Shaw

**Affiliations:** 1 Centre for Pharmacy Postgraduate Education, School of Health Sciences, Division of Pharmacy and Optometry, Faculty of Biology, Medicine and Health, The University of Manchester, Stopford Building (1st Floor), Oxford Road, Manchester, M13 9PT England; 2 Division of Pharmacy and Optometry, Faculty of Biology, Medicine and Health, The University of Manchester, Stopford Building (1st Floor), Oxford Road, Manchester, M13 9PT England

**Keywords:** Assessment tools, Consultation skills, Interrater reliability, Intrarater reliability, Person-centred communication, Pharmaceutical Care

## Abstract

**Background:**

Demonstrating a person-centred approach in a consultation is a key component of delivering high-quality healthcare. To support development of such an approach requires training underpinned by valid assessment tools. Given the lack of a suitable pharmacy-specific tool, a new global consultation skills assessment tool: the medicines related-consultation assessment tool (MR-CAT) was designed and tested.

**Aim:**

This study aimed to test the validity and reliability of the MR-CAT using psychometric methods.

**Method:**

Psychometric testing involved analysis of participants’ (n = 13) assessment of fifteen pre-recorded simulated consultations using the MR-CAT. Analysis included discriminant validity testing, intrarater and interrater reliability testing for each of the five sections of the MR-CAT and for the overall global assessment of the consultation. Analysis also included internal consistency testing for the whole tool.

**Results:**

Internal consistency for the overall global assessment of the consultation was good (Cronbach’s alpha = 0.97). The MR-CAT discriminated well for the overall global assessment of the consultation (*p* < 0.001). Moderate to high intrarater reliability was observed for the overall global assessment of the consultation and for all five sections of the MR-CAT (rho = 0.64–0.84) in the test–retest analysis. Moderate to good interrater reliability (Kendall’s W = 0.68–0.90) was observed for the overall global assessment of the consultation and for all five sections of the MR-CAT.

**Conclusion:**

The MR-CAT is a valid and reliable tool for assessing person-centred pharmacist’s consultations. Moreover, its unique design means that the MR-CAT can be used in both formative and summative assessment.

## Impact statements

- Validated tools for assessing person-centred consultation skills are important for identifying competence of pharmacists as they move into more clinical roles.
- Adopting the MR-CAT as the chosen consultation skills assessment tool for undergraduate and postgraduate pharmacist education in the UK would establish familiarity with advanced person-centred skills for pharmacists and patients making patient partnership and patient autonomy more commonplace.
- Since completion of this study, the MR-CAT has been used in the practice setting to assess over 4000 pharmacists’ consultations as part of a vocational education programme in England. Using a validated assessment tool has contributed to establishing a robust and credible learning programme.

## Introduction

The last decade has seen a transition in the scope of pharmacy practice with a significant shift towards clinical service provision [1–7]. In England, publication of the Long Term Plan in 2019 signalled a clinical future for pharmacy within the National Health Service (NHS) [8]. Today pharmacists are embedded within general practice and care home settings with approximately eleven percent qualified as independent prescribers in the UK [9]. Pharmacists in England play a key role in the provision of clinical services in the community. Examples include the New Medicine Service which involves a consultation with a patient and the NHS urgent care service, which makes it quicker and easier for patients to access advice or treatment [10, 11]. Although development of clinical roles for pharmacists in other countries varies, a common goal of enabling pharmacists to expand their scope of practice and work collaboratively across healthcare systems is emerging [1, 12, 13].

A cornerstone of global pharmacy strategy and policy is the need to develop effective person-centred professionals who are equipped to empower patients as partners in their healthcare and engage in shared-decision making [1, 12, 14–16]. Professional and regulatory bodies have recognised this culture change by placing person-centred care at the heart of pharmacy standards and policy [17–20].

Developing good consultation technique lies at the heart of person-centred care and may lead to improved health outcomes for patients [21–25]. Historically pharmacists were trained as experts in medicines taking a product-centric approach [26]. The concept of patient counselling in relation to medicines is now out-dated and a contradiction to person-centred practice [16, 27].

The need to develop effective person-centred consultation skills as part of pharmacists’ initial education and training and at postgraduate level has been recognised as a priority [28–30]. In England, the national Consultation Skills for Pharmacy Practice programme was developed in 2014 in response [31]. Although undergraduate level training has not kept pace, newly implemented initial education and training standards in the UK have a strong focus on person-centred care [32].

Assessment of consultation performance is noted as the most challenging element of training [33, 34]. Standardised assessment criteria in the form of assessment tools, which allow learners to practise and support constructive feedback, are key to skill development [33, 34]. A lack of validated pharmacy specific consultation assessment tools has led to the exploration of non-pharmacy tools [28, 35–37]. Although deemed suitable for use, many have not been validated for a pharmacy context and were developed prior to the increased emphasis on shared decision-making [28, 35, 36]. The Medication Related Consultation Framework (MRCF) which is widely used in pharmacy consultation skills development and assessment was developed in 2011 [37, 38]. A review of consultation tools to promote the delivery of person-centred consultations in pharmacy recommended revision of the MRCF to satisfy the multi-faceted elements of a true patient-centred consultation [39]. The overall conclusion from the review was that suitable tools are needed to meet the requirements of a holistic patient-centred consultation in a practice setting [39].

As a result, a new global assessment tool; the medicines related consultation assessment tool (MR-CAT) was developed by two members of the research team (referred to as the developers). The MR-CAT is designed to be used by healthcare professionals to assess pharmacists’ consultations; it is not designed as a tool, for patients to assess the consultation. The structure of the MR-CAT (See Table 1) is based on the Calgary-Cambridge model (Initiating the session, Gathering information, Explanation and planning, Closing the session) and an additional section on behaviours which underpin the consultation [21]. The inclusion of shared decision making in the MR-CAT in place of explanation and planning and related behaviours, recognises the shift towards personalised care [40]. Within each section of the MR-CAT there are three levels of practice: below expectations, competent and excellent.

**Table 1:** The descriptors for the five sections of the medicines related-consultation assessment tool (MR-CAT) and the overall global assessment of the consultation for the three levels of practice

Below expectations	Competent	Excellent
1) *INTRODUCTION – How well did the pharmacy professional introduce the consultation and build initial rapport?*		
Vague introduction Fails to establish initial rapport with the patient Establishes the reason for the consultation but no attempt to explore what the patient wants from the consultation **Where other people are present in the consultation** pharmacy professional pays little or no attention to the patient and focuses on the other people present	Clear introduction Pharmacy professional introduces themselves by name and confirms the patient’s identity Attempts to build initial rapport but could improve by applying more welcoming approach and more open body language Establishes the reason for the consultation and explores what the patient wants from the consultation **Where other people are present in the consultation** pharmacy professional establishes who the patient is and who acknowledges them. Focuses the communication on the patient and the other people present	Clear introduction Pharmacy professional introduces themselves by name, explains their role and confirms the patient’s identity Welcoming introduction with open body language which helps to build initial rapport Establishes the reason for the consultation, explores what the patient wants from the consultation and reaches mutual agreement on the purpose and aims of the consultation **Where other people are present in the consultation** pharmacy professional establishes clearly who the patient is and the relationship to other people present. Manages the consultation effectively with all parties with the main focus on communicating with the patient
2) *GATHERING INFORMATION AND IDENTIFYING PROBLEMS – How well did the pharmacy professional identify the patient’s medicines and/or health needs?*		
Demonstrates a closed approach to information gathering with limited opportunity for the patient (or others present) to offer their ideas, concerns and expectations. Poor demonstration of active listening Medicines focused with no exploration of external factors (social, etc.) Patient’s agenda is not explored as the discussion evolves or not acknowledged. Discussion remains pharmacy professional centered	Applies open and closed approaches to exchange information (involving all people present). May benefit from a more open approach to active listening Establishes patient’s ideas, concerns and expectations. Could benefit by exploring these further Identifies external factors which influence health and medicines but may benefit from exploring further Patient’s agenda is explored as the discussion evolves. Could improve by incorporating and balancing the patient’s agenda with the pharmacy professional’s agenda to demonstrate partnership	Applies an open approach to encourage exchange of information (involving all people present). Demonstrates active listening Establishes understanding and explores patient’s ideas concerns and expectations Uses a holistic approach to explore and discuss external factors which may influence health and medicines use Encourages patient to be an equal partner in the discussion. Patient’s agenda is fully explored and differences between the patient’s agenda and the pharmacy professional’s agenda are acknowledged and discussed
3) *SHARED DECISION-MAKING – How well did the pharmacy professional engage the patient in establishing and taking ownership of a management plan?*		
Demonstrates a counselling or ‘telling’ approach. Pharmacy professional directed decisions and no discussion of options ‘What you need to do is …’ Pharmacy professional centered management plan with little/no negotiation with patient (or others present)	Works in partnership with the patient (and other people present), to discuss options and negotiate a mutually acceptable plan that respects the patient’s agenda and preference for involvement Summarises the management plan clearly and concisely but could benefit by checking understanding of the plan with the patient (and others present)	Works in partnership with patient (and other people present), to discuss options Whenever possible, adopts plans that respect the patient’s autonomy. When there is a difference of opinion the patient’s autonomy is respected and a positive relationship is maintained Summarises the management plan clearly and concisely and checks understanding of, and agreement to the plan with the patient (and others present)
4)*CLOSURE – How well did the pharmacy professional negotiate an effective closure to the consultation including discussing safety netting strategies?*		
Concludes the consultation abruptly with no safety net or opportunity for patient (or others present) to ask further questions	Offers a safety net and the opportunity for further questions Clear safety net plan described but could benefit from more input from patient (or others present)	Checks expectations of outcomes and next steps with the patient (and others present) Agrees a safety net plan and ensures all questions from the patient (and others present) are addressed
5)*CONSULTATION BEHAVIOURS – Overall summary of consultation behaviours which forms the structure of the consultation, power versus partnership and therapeutic relationship*		
Overall the consultation structure and discussion is led by the pharmacy professional’s agenda and is one directional Language, tone, body language and attitude may reflect signs of hierarchy from the pharmacy professional Pharmacy professional focused on their own goals with little opportunity for patient (or others present) to contribute Use of jargon or inappropriate language Outcomes are pharmacy professional-centered	Clear structure although may appear rigid due to pharmacy professional addressing their own agenda before that of patient (or others present) Overall good balance of discussion with patient (and others present) offered the opportunity to contribute Good demonstration of active listening skills although may disconnect at points to write notes without pause in discussion Only minor points of jargon or inappropriate language Outcomes are person-centered but may be led by the pharmacy professional	Structure is clear and pharmacy professional summarises to guide the discussion whilst allowing flexibility for the patient’s agenda Overall a balanced equal discussion is established between pharmacy professional, patient (and others present) to demonstrate partnership. Patient (and others present) engage throughout Good demonstration of active listening skills, empathy and appropriate language for the patient Outcomes are negotiated and person-centered
*OVERALL GLOBAL ASSESSMENT OF THE CONSULTATION*		
Fails to demonstrate relevant criteria Pharmacy professional-centered	Demonstrates criteria at a competent level Person-centered	Demonstrates criteria to a high level Person-centered

The developers used the Consultation skills for pharmacy practice: practice standards for England [41] to identify key descriptors summarising the expected skills and behaviours for each of the five sections of the MR-CAT and the overall global assessment of the consultation for below expectations, competent and excellent (See Table 1).

### Aim

This study aimed to test the validity and reliability of the MR-CAT using psychometric methods.

### Ethics approval

The study was approved on 23.08.2018 by the University of Manchester Proportionate Research Ethics Committee reference number 2019–4620-11,787.

## Method

### Production of recordings of simulated consultations

The research team created video recordings of simulated pharmacists’ consultations. Medical actors played the part of patients. The simulated consultations focused on long-term conditions and acute presentations in a primary care setting, for example medication review in a care home, post-discharge asthma review, type-2-diabetes medication review, urinary tract infection and knee pain. The pre-recorded simulated consultations were independently assessed by two members of the research team using the five sections of the MR-CAT and an overall global assessment of the consultation was determined for each recording**.** Fifteen recordings were then used in the validation study covering the three levels of practice for the overall global assessment of the consultation: Below expectations (n = 5), competent (n = 5) and excellent (n = 5)**.** Each recording was given a unique identification number. A further three simulated consultations were recorded to train participants in how to use the MR-CAT.

### Participants and training

Educators involved in training pharmacists to work in advanced practice roles who had also completed prior consultation skills training were invited to participate in the study. These participants (now referred to as ‘raters’) completed training to familiarise them with the MR-CAT and to learn how to use the tool.

Following this, raters independently viewed and assessed the three simulated consultations which had been created for training purposes. Raters then attended a second session where they discussed their rating of each of the recordings and their rationale for a rating to ensure all raters understood how to use the MR-CAT.

### Rating of pre-recorded simulated consultations using the MR-CAT

After completing the training, raters took part in a first round of data collection (January 2020). Raters independently assessed the 15 pre-recorded simulated consultations as below expectations, competent or excellent against the five sections of the MR-CAT and then assigned an overall global assessment of the consultation (below expectations, competent or excellent) based on their ratings for the five sections. Raters were blinded to the levels of practice assigned by the research team to each of the recordings. Raters submitted their ratings for the five sections of the MR-CAT and the overall global assessment of the consultation via an online survey tool. There was a separate survey for each recording to prevent raters from comparing recordings before submitting their ratings. Raters were prevented from accessing the survey after they had submitted ratings so that they could not change or view their ratings after they had been submitted.

The second round of data collection was performed eight weeks after the first round of data collection (March 2020). A sub-sample (n = 6) of the original 15 recordings was used to establish intrarater reliability (test–retest analysis). The six recordings (two below expectations, two competent and two excellent) were given a different unique identification number in the second round and raters were blinded to the levels of practice. Raters independently assessed each recording and submitted their ratings for the five sections of the MR-CAT and the overall global assessment of the consultation via an online survey tool as in the first round.

### Data analysis

Data were downloaded from the survey platform and subsequently analysed using Statistical Package for Social Sciences (SPSS) version 25 database (SPSS Inc., Chicago IL) and STATA for statistics and data management version 14 (StataCorp, College Station, TX). A range of statistical tests were used to test discriminant validity, intrarater and interrater reliability for each of the five sections of the MR-CAT and for the overall global assessment of the consultation. Analysis also included internal consistency testing for the whole tool. The two-tailed p-value was considered significant at *p*-value < 0.05.

Initially, the Cronbach’s alpha test for scales was used to evaluate the internal consistency or how closely the five sections of the tool are related as a group. The overall global assessment of the consultation rating and each of the section ratings (below expectations, competent and excellent) of the MR-CAT were entered into the analysis. To determine internal consistency, a Cronbach’s alpha greater than 0.7, was taken to indicate high internal consistency.

To explore the extent to which the MR-CAT could discriminate between consultations that were below expectations, competent or excellent a Kruskal–Wallis test, with post-hoc Wilcoxon rank sum analysis, was used. This compared raters’ overall global assessment of consultations that had a *priori* been classified as below expectations, competent or excellent using the mean and standard deviation with the rating of the raters for statistical differences between grouped consultation types.

The degree to which raters awarded similar ratings when observing the same consultations was investigated (inter-rater reliability). Each rater’s ratings for the five sections of the MR-CAT and the overall global assessment of the consultation were ranked across the 15 simulated consultations. Kendall’s coefficient of concordance was calculated to assess the degree of agreement between raters’ ranked ratings at each level of practice.

The extent to which raters produced consistent ratings when applying the MR-CAT to the same simulated consultation at two time points was investigated (intrarater reliability). The test–retest values were produced at Time 1 (original test of tool) and Time 2 (eight weeks after the original test) and compared. Spearman’s correlation coefficients (rho) were calculated for each section of the MR-CAT using rank orders of ordinal data.

## Results

13 pharmacy educators participated in the study.

### Internal consistency

The Cronbach’s alpha was very good at 0.97, demonstrating internal consistency of the MR-CAT and very good correlation between the sections of the MR-CAT and the overall global assessment of the consultation (all rated at r = 1.00).

### Discriminant validity

**Fig. 1:**
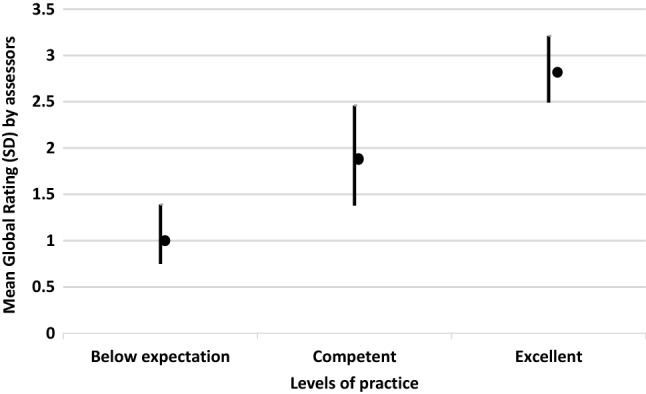
Differences between mean ratings on the global assessment level

Analysis of the raters’ mean ratings for the overall global assessment of the consultation were found to discriminate between the three levels of practice (below expectations, competent and excellent) (Kruskal–Wallis Chi-square = 128.71; df = 2; *p*-value < 0.001) (See Fig. 1).

The post-hoc Wilcoxon rank sum analysis revealed that there were significant differences between each level of practice (z score < -8.33; *p*-value < 0.001 for all three levels of practice). Variation in raters’ mean ratings for the overall global assessment of the consultation was biggest for the competent level of practice (SD 0.54); consensus was greatest regarding the below expectations (or fail) level of practice (SD 0.32).,

### Interrater reliability

Interrater reliability for all five sections of the MR-CAT and the overall global assessment of the consultation was good (Kendall’s W 0.80–0.90) apart from the introduction section of the MR-CAT where there was moderate agreement (Kendall’s W 0.68). Agreement was highest for the overall global assessment of the consultation and for the shared-decision making and consultation behaviours sections of the MR-CAT and was significant at *p*-value < 0.05 and 14 degrees of freedom for all five sections of the MR-CAT and for the overall global assessment of the consultation (see Table 2).

**Table 2:** Interassessor reliability (Kendall's W score) and intraassessor (test—retest) reliability score (Spearman's rho) for each section of the MR-CAT

Section of MR-CAT	Kendall’s W score	*p*-value	Spearman’s rho	*p*-value
Global assessment of consultation	0.85	< 0.001	0.76	< 0.001
Introduction	0.68	< 0.001	0.70	< 0.001
Gathering information and identifying problems	0.80	< 0.001	0.64	< 0.001
Shared-decision making	0.90	< 0.001	0.84	< 0.001
Closure	0.80	< 0.001	0.76	< 0.001
Behaviours	0.84	< 0.001	0.77	< 0.001

### Intrarater (test–retest) reliability

There was moderate to high intrarater reliability for all five sections of the MR-CAT and for the overall global assessment of the consultation (rho 0.64–0.84) in the test–retest analysis. Two-tailed *p*-value was considered significant at *p*-value < 0.05 (see Table 2). Raters 8 and 13 did not take part in this analysis.

## Discussion

### Key findings

Using psychometric methods, this study sought to test the validity and reliability of the MR-CAT. Our findings suggest that the MR-CAT is a valid and reliable instrument, that is capable of discriminating between different levels of consultation practice (below expectations, competent and excellent). This differentiates the MR-CAT from previous tools. The PharmaCAT uses a rating scale ranging from poor to excellent practice, whilst the MRCF offers descriptors of key skills and behaviours with the option to select yes or no if observed in a consultation [36, 37]. The MR-CAT design includes descriptors with clear definition of specific skills and behaviours which demonstrate each level of practice, consistent with the format used in the consultation tool used in medical education [36].

Interrater reliability was highest for the shared decision making and consultation behaviours sections of the MR-CAT and the global assessment rating. While, on the other hand, interrater agreement was only moderate for the introduction section of the MR-CAT; this finding is similar to other studies [37].

### Summative assessment: avoiding failure to fail

When considering discriminant validity and the three levels of practice there was highest agreement in rater ratings for the ‘below expectations’ level of practice. This is important when using a tool for summative assessment (assessment of learning) as it suggests greatest consensus over what is a fail. Failing to fail underperforming students is a well-documented problem, which has serious implications for patient safety and professional competence [42–45]. One of the barriers cited as contributing to failure to fail is a lack of certainty and/or clarity around expected standards of performance particularly if assessors are inexperienced or lack confidence [42–45]. The use of criterion-referenced frameworks is recommended to promote clarity and fair and consistent treatment of learners and has been incorporated within the MR-CAT [45]. The inclusion of descriptors within the MR-CAT provides clear expectations of performance for learners and assessors for different levels of practice which assures fairness and equity in the assessment process and has the potential to help address the issue of failing to fail.

### Formative assessment: preparing to pass

Theories which support the development of communication skills include practice in the workplace and reflective theory [46–49]. The MR-CAT has the potential to facilitate such learning in a practice setting because the global structure and descriptors support learners and assessors to differentiate between levels of practice and conceptualise what good consultation skills and behaviours are. Reflection is promoted by identification of strengths and areas for development. This in turn facilitates timely and personalised feedback in the consultation, a key component of skill development [33, 36, 50, 51] and further supports the potential utility of the MR-CAT for formative assessment (assessment for learning). The advantage this brings is that methods of summative assessment which also have a formative role are better than those that do not [52].

### Advantages of global assessment tool design

Our findings provide assurance of the MR-CAT design as a global assessment tool that is capable of discriminating between different levels of pharmacist’s consultation practice (below expectations, competent and excellent). Tools containing more detailed skill elements whilst supporting a good structure and understanding of the consultation may hinder their application in practice [39]. Validation of the MRCF identified some inconsistencies in rater ratings of individual consultation behaviours within the discrete elements of the tool [37]. Given evidence that global assessment tools should be used in preference to a checklist approach to develop competence and improve professional authenticity it is likely that MR-CAT will be useful for assessing capability and support personal consultation style which adapts to the needs of the individual patient [53].

### Limitations of the study

Limitations of the study include its small sample size (n = 13). However, the participants were educators involved in training pharmacists to work in advanced practice roles who had also completed prior consultation skills training. Therefore, the sample was very homogenous. A similar study which used psychometric testing to validate the MRCF also used a small homogenous sample (n = 10) which has been shown to be acceptable [37].

There was moderate to high intrarater reliability for all five sections of the MR-CAT and the overall global assessment of the consultation. The authors acknowledge the potential for memory bias due to the raters being presented with six of the original 15 simulated recorded consultations for the test–retest analysis. Human memory is a complex and broad concept [54]. Eight-weeks was chosen for the test–retest analysis because this was the same length of time between the first and second round of data collection in the MRCF validation study which was previously accepted as a methodologically robust approach [37].

Internal consistency for the overall global assessment of the consultation was good (Cronbach’s alpha = 0.97). A high Cronbach’s alpha > 0.95 suggests that there is collinearity among the items tested and may suggest redundancy. The items tested are the descriptors for each of the five sections of the MR-CAT (see Table 1) which assess different sections of the consultation. On this basis, it is unlikely that the high Crohnbach's alpha suggests redundancy. The most likely explanation for the high Cronbach’s alpha is if a pharmacist performs well in one section of the consultation, they are more likely to perform well in the other sections of the consultation. This is observed by the standard deviation (SD) of the overall global assessment of the consultation ratings given for each pre-recorded simulated videoed consultation (see Fig. 1). Those rated excellent and below expectations have very small SDs, indicating little variation in the rating. However, those rated competent have a larger SD suggesting that there is more variation in the assessment of a competent consultation.

While this validation study supports MR-CAT’s psychometric properties, this tool has so far only been tested using simulated consultations. There remains a need to undertake further research to test the usability and utility of the MR-CAT in a practice setting, possibly using a similar approach to ours which is widely accepted [55, 56]. Moreover, the pre-recorded simulated video consultations were situated in simulated general practice and care homes settings and were conducted by pharmacists. Therefore, further research would be required to assure its utility across other sectors of practice and for pharmacy technicians’ consultations.

## Conclusion

The need for all healthcare professionals to demonstrate a person-centred approach in a consultation is imperative to quality of care across the health system and to providing personalised care. Developed specifically for the assessment of pharmacists’ consultation skills by observation of practice, the MR-CAT has been validated as a summative assessment tool which is useful to support pharmacists in advanced patient-facing roles. The ability of the MR-CAT to discriminate well between levels of practice for all five sections of the MR-CAT and for the overall global assessment of the consultation with specific descriptors of practice enables pharmacists to identify strengths and areas for development. This strengthens MR-CATs position as a formative assessment tool by enabling constructive feedback and reflection on practice.
