# Correction to: Reliability and validity testing of the medicines related - consultation assessment tool for assessing pharmacists’ consultations

**DOI:** 10.1007/s11096-023-01549-1

**Published:** 2023-03-15

**Authors:** Helen Middleton, Lesley Grimes, Sarah C. Willis, Douglas Steinke, Matthew Shaw

**Affiliations:** 1 Centre for Pharmacy Postgraduate Education, School of Health Sciences, Division of Pharmacy and Optometry, Faculty of Biology, Medicine and Health, The University of Manchester, Stopford Building (1st Floor), Oxford Road, Manchester, M13 9PT England; 2 Division of Pharmacy and Optometry, Faculty of Biology, Medicine and Health, The University of Manchester, Stopford Building (1st Floor), Oxford Road, Manchester, M13 9PT England

**Correction to: International Journal of Clinical Pharmacy** **https://doi.org/10.1007/s11096-022-01489-2**

In the original publication of the article, the author name “James DH” is incorrectly abbreviated as “Higman JD” in the 37^th^ reference.

The correct reference is “Abdel-Tawab R, James DH, Fichtinher A, et al. Development of the medication-related consultation framework (MRCF). Patient Educ Couns. 2011;2011(83):451–7.”

The original article has been corrected.

